# Influenza virus infection among pediatric patients reporting diarrhea and influenza-like illness

**DOI:** 10.1186/1471-2334-10-3

**Published:** 2010-01-07

**Authors:** Charisma Dilantika, Endang R Sedyaningsih, Matthew R Kasper, Magdarina Agtini, Erlin Listiyaningsih, Timothy M Uyeki, Timothy H Burgess, Patrick J Blair, Shannon D Putnam

**Affiliations:** 1 Naval Medical Research Unit No. 2, Jakarta, Indonesia; 2 Center for Biomedical and Pharmaceutical Research and Development, National Institute of Health Research and Development, Jakarta, MOH Indonesia; 3 Epidemiology and Prevention Branch, Influenza Division, Centers for Disease Control and Prevention, Atlanta, Georgia, USA

## Abstract

**Background:**

Influenza is a major cause of morbidity and hospitalization among children. While less often reported in adults, gastrointestinal symptoms have been associated with influenza in children, including abdominal pain, nausea, vomiting, and diarrhea.

**Methods:**

From September 2005 and April 2008, pediatric patients in Indonesia presenting with concurrent diarrhea and influenza-like illness were enrolled in a study to determine the frequency of influenza virus infection in young patients presenting with symptoms less commonly associated with an upper respiratory tract infection (URTI). Stool specimens and upper respiratory swabs were assayed for the presence of influenza virus.

**Results:**

Seasonal influenza A or influenza B viral RNA was detected in 85 (11.6%) upper respiratory specimens and 21 (2.9%) of stool specimens. Viable influenza B virus was isolated from the stool specimen of one case. During the time of this study, human infections with highly pathogenic avian influenza A (H5N1) virus were common in the survey area. However, among 733 enrolled subjects, none had evidence of H5N1 virus infection.

**Conclusions:**

The detection of influenza viral RNA and viable influenza virus from stool suggests that influenza virus may be localized in the gastrointestinal tract of children, may be associated with pediatric diarrhea and may serve as a potential mode of transmission during seasonal and epidemic influenza outbreaks.

## Background

Influenza is a major cause of morbidity and hospitalization among children [1-5]. Complications of influenza among children are well-documented, including bronchiolitis, pneumonia, as well as neurological manifestations such as acute encephalopathy and encephalitis [6]. While less often reported in adults, gastrointestinal symptoms have been associated with influenza in children, including abdominal pain, nausea, vomiting, and diarrhea [7,8]. One study reported detection of influenza viral RNA in rectal swabs from two infants confirmed with influenza disease [9].

Diarrhea has been reported among confirmed influenza A (H5N1) cases [10,11], in some cases preceding lower respiratory tract involvement [12-14]. Detection of influenza A (H5N1) viral RNA and the isolation and identification of influenza A (H5N1) virus, in cell culture, has been reported from rectal swab, stool, and enteral tissue specimens from fatal, confirmed A (H5N1) cases [15-17]. Despite the recognized clinical association between these enteric manifestations and influenza, limited data are available about whether influenza viruses are present in the gastrointestinal tract of children with influenza-like illness.

Influenza activity occurs year-round in Indonesia, with increased activity during the rainy season (November through April) and peaking in December-January [18]. In addition to seasonal influenza, Indonesia has reported the highest number of influenza A (H5N1) cases worldwide (N = 139, December 2008; http://www.who.int), with a case fatality proportion reported between 75% and 80% [19,20]. The epidemiology of human cases of influenza A (H5N1) in Indonesia shows that 96% of the cases occurred in persons < 40 years of age, 24% < 10 years of age, with a median age 18.5 years; nearly two-thirds were classified as rural inhabitants [19].

The primary objective of this study was to determine the prevalence of seasonal influenza A and B viruses, and avian influenza A (H5N1) virus, among children seeking medical care with acute influenza-like illness (ILI) and diarrhea in Indonesia.

## Methods

### Study site and population

Children < 6 years of age with concurrent diarrhea and acute influenza-like illness presenting at any of 12 study sites (Indonesian Pediatric Diarrhea Network) between September 2005 and April 2008 were eligible for enrollment. Including in the Indonesian Pediatric Diarrhea Network were established study sites located in Jakarta, West Java; Yogyakarta, Central Java; Denpasar, Bali; Mataram, Lombok; Makassar, Sulawesi and Medan, North Sumatra. Stool and upper respiratory (nasal swab and throat swab) specimens were collected from each participant after informed consent was obtained. Nasal swab specimens were collected from the mid-turbinate region and the swab was placed in 2-3 ml of viral transport media (VTM). For throat swabs, both tonsils and the posterior pharynx were swabbed vigorously, and the swab placed in 2-3 ml of VTM. Collected specimens were placed into viral transport media and shipped on ice packs to the laboratory via air or ground courier, within 24 hours after collection. Upon arrival specimens were stored at -70°C until testing. Influenza-like illness was defined as acute fever (> 38.0°C) with cough or sore throat; diarrhea was defined as three or more loose/liquid stools or two or more loose/liquid stools with one or more associated enteric symptoms (e.g., nausea, vomiting, abdominal cramps, or bloody stool) during any 24 hour period of the current episode. Seasonality was defined as either 'rainy' season (November through April) or 'dry' season (May through October) for this study.

### Laboratory testing

Specimen testing was conducted at a central laboratory in Jakarta, Indonesia. RNA was extracted from VTM of throat and nasal swab specimens and stool specimens re-suspended in a 10% solution of PBS using the QIAmp viral RNA mini kit (Qiagen, Hilden, Germany). Identification of influenza type and influenza A subtypes was conducted using multiplex nested reverse-transcription polymerase chain reaction (RT-PCR) as previously described [21,22]. Based on RT-PCR results, a random sample of positive clinical specimens was selected for viral isolation; a random sample of negative specimens was sent for viral culture as well. Briefly, specimens were inoculated onto Madin-Darby canine kidney (MDCK) tissue cells with an aliquot of clinical specimens and incubated for 7 days; indirect immunofluorescence assay (IFA) using specific influenza labeled antibodies was employed to confirm the presence of influenza.

### Data analysis

All data was double-entered into a MS Access (Microsoft Corp., Redmond, Washington, USA) database and statistical testing was conducted using SAS 9.1.13 (SAS Institute, Cary North Carolina, USA).

### Ethical Considerations

This study was reviewed and approved by the Ethical Commission of the National Institute of Health Research and Development, Indonesian Ministry of Health, Jakarta, Indonesia and by the Institutional Review Board for the ethical conduct of research on human subjects at the U.S. Naval Medical Research Unit No. 2, Jakarta, Indonesia.

## Results

### Characteristics of patients

From September 2005 through April 2008, 733 pediatric patients (< 6 years of age) with diarrhea and influenza-like illness were enrolled. Each participant provided matched clinical specimens (stool, one nasal swab and one throat swab) for laboratory analysis. The median age of participants was 15 months (interquartile range (IQR), 9 - 24 months) and 59.5% (436) were male. The median duration of the enrollment diarrhea episode was 1 day (IQR, 1 - 2 days). The median duration of current influenza-like illness episode was 2 days (IQR, 1 - 2). Malnutrition and dehydration among the study participants, subjectively assigned by the enrolling physician, was 7.4% and 25.8%, respectively. Diarrhea severity was subjectively defined by the physician and 71.5% of the study participants were assigned as having either 'mild' or 'moderate' diarrhea. Of the 733 study participants, 80 (10.9%) were hospitalized, 11 (1.5%) tested positive for influenza virus by RT-PCR. Table 1 lists the sampling demographics, signs and symptoms, severity of illness and other covariates, stratified by specimen site.

**Table 1:** Demographics, signs and symptoms of study participants stratified by clinical specimen site (n = 733).

	Stool (N = 15)	**Upper Respiratory**^a^ (N = 79)	Both (N = 6)	Neither (N = 633)
Age^b ^(months)	12 (9 - 15)	19 (10 - 27)	9 (5 - 27)	14 (8 - 23)
Diarrhea duration^b ^(days)	2 (1 - 3)	1 (1 - 2)	1 (1 - 2)	1 (1 - 2)
Influenza-like illness duration^b ^(days)	3 (1 - 4)	2 (1 - 3)	2 (2 - 3)	2 (1 - 3)
Fever duration^b ^(days)	2 (1 - 2)	1 (1 - 2)	1 (1 - 3)	1 (1 - 2)
				
Gender^c^				
Male	10 (66.7%)	44 (55.7%)	3 (50.0%)	379 (59.9%)
Female	5 (33.3%)	35 (44.3%)	3 (50.0%)	254 (40.1%)
				
Age^c ^(months)				
0-5	0 (0.0%)	2 (2.5%)	2 (33.3%)	65 (10.3%)
6-11	5 (33.3%)	24 (30.4%)	1 (0.0%)	176 (27.8%)
12-23	8 (53.3%)	23 (29.1%)	0 (0.0%)	234 (37.0%)
24-35	1 (6.7%)	16 (20.3%)	3 (50.0%)	92 (14.5%)
36-46	0 (0.0%)	7 (8.9%)	0 (0.0%)	37 (5.8%)
47-71	1 (6.7%)	7 (8.9%)	0 (0.0%)	29 (4.6%)
				
Diarrhea Severity^c^				
Mild	4 (26.7%)	29 (36.7%)	2 (33.3%)	166 (26.7%)^d^
Moderate	10 (66.7%)	28 (35.4%)	3 (50.0%)	274 (44.1%)^d^
Severe	1 (6.7%)	21 (26.6%)	1 (16.7%)	182 (29.2%)^d^
				
Malnutrition^c^	1 (6.7%)	8 (11.3%)^d^	0 (0.0%)^d^	41 (7.0%)^d^
				
Fever^c^	15 (100%)	79 (100%)	6 (100%)	633 (100%)
				
Cough^c^	15 (100%)	76 (96.2%)	6 (100%)	609 (96.2%)
				
Hospitalization^c^	2 (13.3%)	8 (10.1%)	1 (16.7%)	69 (10.9%)

a. Upper respiratory is a composite variable of throat and nasal specimen.

b. Results presented as median (interquartile range)

c. Results presented as number (percent)

d. Percent values reflect missing denominator data

### Influenza detection

Of 733 study participants, influenza virus was detected by RT-PCR in 85 (11.6%) upper respiratory tract specimens and 21 (2.9%) stool specimens. Only six patients had concurrent influenza virus identified from both upper respiratory and stool specimens. Of the 100 patients that tested positive for influenza, influenza A and influenza B virus accounted for 40 (40.0%) and 60 (60.0%), respectively. Of the influenza A viruses identified in upper respiratory specimens, 13 were A (H1N1) and 27 were A (H3N2). Both influenza A and B viruses were detected in stool specimens, including one A (H1N1), three A (H3N2), and 17 B viruses by PCR. In addition to the molecular detection of influenza viral RNA, a subset of randomly selected PCR positive and PCR negative samples (18 and 38, respectively) were submitted for viral culture with a total of 15 (26.8%) positive from upper respiratory specimens. From 25 stool specimens (13 positive and 12 negative by PCR) there was one (4.0%) positive. There were no PCR negative samples that tested positive by viral culture.

## Discussion

To our best of our knowledge, this is the largest study investigating influenza virus detection in the gastrointestinal tract of children with influenza-like illness and diarrhea. This study found evidence of influenza viral RNA in 21 (2.9%) stool specimens collected from pediatric patients aged < 6 years presenting with influenza-like illness (ILI) and diarrhea, thus confirming the findings of a very small study in young children aged 0 - 23 months [9]. The successful recovery of viable influenza B virus from the stool of a single case was notable; it is not clear whether detection of influenza virus in the stool represents active infection of the gastrointestinal tract or swallowed virus from an upper respiratory tract infection. It is possible that influenza viruses might bind to α2,6 sialic acid receptors in the human gastrointestinal tract, and infect and actively replicate within cells in the gastrointestinal tract [23], similar to influenza viruses replication in avian species. However, two studies did not find any evidence for human influenza virus receptors in epithelial cells of the intestinal tract [24,25]. Of interest, influenza virus was the only pathogen detected in several cases of diarrheic children with ILI. Studies are being planned to further confirm these findings and investigate the gastrointestinal activity of influenza virus among influenza positive patients.

We did not detect highly pathogenic avian influenza A (H5N1) viral RNA from any clinical specimens, even though fever and diarrhea have been reported as presenting signs and symptoms in some H5N1 patients [13,14,19]. During the study period, Indonesia reported the highest number of H5N1 human cases of any country http://www.who.int, and H5N1 is endemic among poultry in Indonesia, human infection with H5N1 virus continues to be a rare event.

The low yield of influenza virus detected from stool could be due to several factors. It is possible that influenza virus infection and viral shedding in the gastrointestinal tract may be truly low in children with influenza. It is also possible that detection of influenza virus in stool or rectal swab specimens, either by RT-PCR or viral culture, may have been reduced due to inhibitory material present in the gastrointestinal tract and stool. Alternatively, the amount of time, transport time, between specimen collection and inoculation may have rendered the viruses non-viable and subsequently had degradation of influenza target. Additional studies are warranted to further investigate influenza virus detection, including the swine-origin influenza A virus [26,27], in stool specimens of acute ILI patients with and without diarrhea. In addition, studies that further examine influenza virus binding to intestinal tract epithelial tissue, and replication of influenza viruses within the gastrointestinal tract are needed.

## Conclusions

The detection of influenza viral RNA and viable influenza virus from stool suggests that influenza virus may be localized in the gastrointestinal tract of children, may be associated with pediatric diarrhea and may serve as a potential mode of transmission during seasonal and epidemic influenza outbreaks.

## Competing interests

The authors declare that they have no competing interests.

## Authors' contributions

CD - laboratory supervision and testing, data analysis, writing manuscript. ERS - Study design, review and corrections of manuscript. MRK - Data analysis, writing manuscript, revisions to manuscript. MA - study oversight, data management and analysis. EL - Study oversight and supervision of laboratory testing. TMU - Study design, review and corrections of manuscript. THB - Study design, review and corrections of manuscript. PJB - Study design, review and corrections of manuscript. SDP - study principal investigator (Sept 2005 - April 2008), study design and implementation, data management, data analysis, writing manuscript. All authors read and approved the final manuscript.

## Pre-publication history

The pre-publication history for this paper can be accessed here:

http://www.biomedcentral.com/1471-2334/10/3/prepub

## References

[B1] Coffin SE Zaoutis TE Rosenquist AB Heydon K Herrera G Bridges CB Watson B Localio R Hodinka RL Keren R Incidence, complications, and risk factors for prolonged stay in children hospitalized with community-acquired influenza Pediatrics 2007 119 740 748 10.1542/peds.2006-2679 17403845

[B2] Louie JK Schechter R Honarmand S Guevara HF Shoemaker TR Madrigal NY Woodfill CJ Backer HD Glaser CA Severe pediatric influenza in California, 2003-2005: implications for immunization recommendations Pediatrics 2006 117 e610 618 10.1542/peds.2005-1373 16585278

[B3] Bhat N Wright JG Broder KR Murray EL Greenberg ME Glover MJ Likos AM Posey DL Klimov A Lindstrom SE Influenza-associated deaths among children in the United States, 2003-2004 N Engl J Med 2005 353 2559 2567 10.1056/NEJMoa051721 16354892

[B4] Poehling KA Edwards KM Weinberg GA Szilagyi P Staat MA Iwane MK Bridges CB Grijalva CG Zhu Y Bernstein DI The underrecognized burden of influenza in young children N Engl J Med 2006 355 31 40 10.1056/NEJMoa054869 16822994

[B5] Schrag SJ Shay DK Gershman K Thomas A Craig AS Schaffner W Harrison LH Vugia D Clogher P Lynfield R Multistate surveillance for laboratory-confirmed, influenza-associated hospitalizations in children: 2003-2004 Pediatr Infect Dis J 2006 25 395 400 10.1097/01.inf.0000214988.81379.71 16645501

[B6] Morishima T Togashi T Yokota S Okuno Y Miyazaki C Tashiro M Okabe N Encephalitis and encephalopathy associated with an influenza epidemic in Japan Clin Infect Dis 2002 35 512 517 10.1086/341407 12173123

[B7] Wang YH Huang YC Chang LY Kao HT Lin PY Huang CG Lin TY Clinical characteristics of children with influenza A virus infection requiring hospitalization J Microbiol Immunol Infect 2003 36 111 116 12886962

[B8] Liou YS Barbour SD Bell LM Plotkin SA Children hospitalized with influenza B infection Pediatr Infect Dis J 1987 6 541 543 3615069 10.1097/00006454-198706000-00011

[B9] Wootton SH Scheifele DW Mak A Petric M Skowronski DM Detection of human influenza virus in the stool of children Pediatr Infect Dis J 2006 25 1194 1195 17133173 10.1097/01.inf.0000245097.95543.11

[B10] Abdel-Ghafar AN Chotpitayasunondh T Gao Z Hayden FG Nguyen DH de Jong MD Naghdaliyev A Peiris JS Shindo N Soeroso S Uyeki TM Update on avian influenza A (H5N1) virus infection in humans N Engl J Med 2008 358 261 273 10.1056/NEJMra0707279 18199865

[B11] Tran TH Nguyen TL Nguyen TD Luong TS Pham PM Nguyen VC Pham TS Vo CD Le TQ Ngo TT Avian influenza A (H5N1) in 10 patients in Vietnam N Engl J Med 2004 350 1179 1188 10.1056/NEJMoa040419 14985470

[B12] Peiris JS de Jong MD Guan Y Avian Influenza Virus (H5N1): a Threat to Human Health Clin Microbiol Rev 2007 20 243 267 10.1128/CMR.00037-06 17428885 PMC1865597

[B13] Apisarnthanarak A Kitphati R Thongphubeth K Patoomanunt P Anthanont P Auwanit W Thawatsupha P Chittaganpitch M Saeng-Aroon S Waicharoen S Atypical avian influenza (H5N1) Emerg Infect Dis 2004 10 1321 1324 15324560 10.3201/eid1007.040415 PMC3323328

[B14] de Jong MD Bach VC Phan TQ Vo MH Tran TT Nguyen BH Beld M Le TP Truong HK Nguyen VV Fatal avian influenza A (H5N1) in a child presenting with diarrhea followed by coma N Engl J Med 2005 352 686 691 10.1056/NEJMoa044307 15716562

[B15] de Jong MD Simmons CP Thanh TT Hien VM Smith GJ Chau TN Hoang DM Chau NV Khanh TH Dong VC Fatal outcome of human influenza A (H5N1) is associated with high viral load and hypercytokinemia Nat Med 2006 12 1203 1207 10.1038/nm1477 16964257 PMC4333202

[B16] Uiprasertkul M Puthavathana P Sangsiriwut K Pooruk P Srisook K Peiris M Nicholls JM Chokephaibulkit K Vanprapar N Auewarakul P Influenza A H5N1 replication sites in humans Emerg Infect Dis 2005 11 1036 1041 16022777 10.3201/eid1107.041313 PMC3371815

[B17] Buchy P Mardy S Vong S Toyoda T Aubin JT Miller M Touch S Sovann L Dufourcq JB Richner B Influenza A/H5N1 virus infection in humans in Cambodia J Clin Virol 2007 39 164 168 10.1016/j.jcv.2007.04.010 17526430

[B18] Beckett CG Kosasih H Ma'roef C Listiyaningsih E Elyazar IR Wuryadi S Yuwono D McArdle JL Corwin AL Porter KR Influenza surveillance in Indonesia: 1999-2003 Clin Infect Dis 2004 39 443 449 10.1086/422314 15356802

[B19] Sedyaningsih ER Isfandari S Setiawaty V Rifati L Harun S Purba W Imari S Giriputra S Blair PJ Putnam SD Epidemiology of cases of H5N1 virus infection in Indonesia, July 2005-June 2006 J Infect Dis 2007 196 522 527 10.1086/519692 17624836

[B20] Kandun IN Wibisono H Sedyaningsih ER Yusharmen Hadisoedarsuno W Purba W Santoso H Septiawati C Tresnaningsih E Heriyanto B Three Indonesian clusters of H5N1 virus infection in 2005 N Engl J Med 2006 355 2186 2194 10.1056/NEJMoa060930 17124016

[B21] Zhang WD Evans DH Detection and identification of human influenza viruses by the polymerase chain reaction J Virol Methods 1991 33 165 189 10.1016/0166-0934(91)90017-T 1939505

[B22] Gu J Xie Z Gao Z Liu J Korteweg C Ye J Lau LT Lu J Gao Z Zhang B H5N1 infection of the respiratory tract and beyond: a molecular pathology study Lancet 2007 370 1137 1145 10.1016/S0140-6736(07)61515-3 17905166 PMC7159293

[B23] Beigel JH Farrar J Han AM Hayden FG Hyer R de Jong MD Lochindarat S Nguyen TK Nguyen TH Tran TH Avian influenza A (H5N1) infection in humans N Engl J Med 2005 353 1374 1385 10.1056/NEJMra052211 16192482

[B24] Sata T Roth J Zuber C Stamm B Heitz PU Expression of alpha 2,6-linked sialic acid residues in neoplastic but not in normal human colonic mucosa. A lectin-gold cytochemical study with Sambucus nigra and Maackia amurensis lectins Am J Pathol 1991 139 1435 1448 1661075 PMC1886452

[B25] Yao L Korteweg C Hsueh W Gu J Avian influenza receptor expression in H5N1-infected and noninfected human tissues Faseb J 2008 22 733 740 10.1096/fj.06-7880com 17925493

[B26] Dawood FS Jain S Finelli L Shaw MW Lindstrom S Garten RJ Gubareva LV Xu X Bridges CB Uyeki TM Emergence of a novel swine-origin influenza A (H1N1) virus in humans N Engl J Med 2009 360 2605 2615 10.1056/NEJMoa0903810 19423869

[B27] Shinde V Bridges CB Uyeki TM Shu B Balish A Xu X Lindstrom S Gubareva LV Deyde V Garten RJ Triple-reassortant swine influenza A (H1) in humans in the United States, 2005-2009 N Engl J Med 2009 360 2616 2625 10.1056/NEJMoa0903812 19423871

